# Effects of Cocooning on Coronavirus Disease Rates after Relaxing Social Distancing 

**DOI:** 10.3201/eid2612.201930

**Published:** 2020-12

**Authors:** Xutong Wang, Zhanwei Du, George Huang, Remy F. Pasco, Spencer J. Fox, Alison P. Galvani, Michael Pignone, S. Claiborne Johnston, Lauren Ancel Meyers

**Affiliations:** The University of Texas at Austin, Austin, Texas, USA (X. Wang, Z. Du, G. Huang, R.F. Pasco, S.J. Fox, L.A. Meyers);; Yale School of Public Health, New Haven, Connecticut, USA (A.P. Galvani); T; he University of Texas at Austin Dell Medical School, Austin (M. Pignone, S. Claiborne Johnston);; Santa Fe Institute, Santa Fe, New Mexico, USA (L. Ancel Meyers)

**Keywords:** respiratory infections, severe acute respiratory syndrome coronavirus 2, SARS-CoV-2, SARS, COVID-19, coronavirus disease, zoonoses, viruses, coronavirus, epidemiology, pandemics, mathematical model, hospital bed capacity

## Abstract

As coronavirus disease spreads throughout the United States, policymakers are contemplating reinstatement and relaxation of shelter-in-place orders. By using a model capturing high-risk populations and transmission rates estimated from hospitalization data, we found that postponing relaxation will only delay future disease waves. Cocooning vulnerable populations can prevent overwhelming medical surges.

In March 2020, cities and states throughout the United States issued social distancing orders to mitigate the coronavirus disease (COVID-19) pandemic (*1*). In response to growing political and economic pressures, the White House and the Centers for Disease Control and Prevention issued guidelines for relaxing such measures on April 16, 2020 (*2*). However, the gating criteria in these guidelines do not include provisions, such as cocooning, to protect vulnerable populations. Residents of long-term care facilities (LTCFs) are particularly vulnerable because of congregate living, shortages in qualified workers, and the need for physical contact between caregivers and residents. In LTCFs, cocooning includes measures to increase staff; cohort residents; test for severe acute respiratory syndrome 2 (SARS-CoV-2), the causative agent of COVID-19; and assess availability of personal protective equipment and other infection control resources (*3*). Among other groups, cocooning involves incentivizing persons with high-risk underlying conditions to remain at home, helping persons experiencing homelessness to social distance, and broadly encouraging hand hygiene and wearing face masks for persons at high risk for severe illness or death and their caregivers (*4*). 

By June 16, 2020, nursing home residents constituted 42.8% (50,919/119,055) of US COVID-19 deaths (*5*). In Austin, Texas, patients in LTCFs represented approximately half the COVID-19 deaths and >20% (81/398) of COVID-19 hospitalizations among persons with known residence (*6*). 

To quantify the need for proactively protecting these vulnerable populations, we projected the effects of relaxation of shelter-in-place orders, with and without additional cocooning measures. We built a granular mathematical model of COVID-19 spread in US cities that incorporates age-specific and risk-stratified heterogeneity in the transmission and severity of COVID-19 (Appendix) (*7*). The model uses 70 stochastic differential equations to track the disease status in 10 subpopulations: low-risk and high-risk persons in each of 5 age groups, 0–4 years, 5–17 years, 18–49 years, 50–64 years, and >64 years of age. We focused on the Austin-Round Rock Metropolitan Statistical Area in Texas, the fastest-growing large city area in the United States, because we provide decision support for city leaders and have access to patient-level COVID-19 hospitalization and death data. 

Persons initially are susceptible to SARS-CoV-2 and infection rates are dependent on age-specific contact rates and prevalence of infection. Upon infection, persons incubate SARS-CoV-2 asymptomatically before progressing to a symptomatic or asymptomatic infectious state. Depending on age and risk group, symptomatic COVID-19 case-patients might be hospitalized and die. To model cocooning of high-risk populations, we reduced the transmission rate to and from persons >64 years of age and in younger high-risk subgroups.

Social distancing began in Austin with school closures on March 14, 2020 and ramped up on March 24, 2020 with a Stay Home–Work Safe order (order 20200324-007; https://www.austintexas.gov). We assumed published values for most model parameters (Table; Appendix) and calibrated the transmission rate before and after the stay-home order based on hospitalization counts (Figure). During March 24–April 23, data suggest that SARS-CoV-2 transmission dropped by 70% (95% CI 45%–100%). If social distancing measures were completely relaxed on May 1, 2020, we estimated that COVID-19 hospitalizations would surpass Austin’s surge capacity of 3,440 beds in 27 (95% CI 16–43) days, on May 28 (Figure). Assuming instead that individual behavior and public health efforts continued to reduce transmission by 75% relative to the stay-home order, hospital surge capacity would be reached after 84 (95% CI 41–137) days, on July 24. When we superimposed cocooning to reduce transmission risk by 125% relative to the stay-home period for 547,474 persons at high risk among the total population of 2,168,316 (Appendix), Austin could avoid hospital surge and reduce cumulative COVID-19 hospitalizations by 62% and deaths by 70% (Appendix Table 1). Postponing relaxation of shelter-in-place measures would not prevent a second pandemic wave but could buy more time to protect vulnerable populations (Appendix Figure 1).

**Table:** Key parameters of a transmission model for coronavirus disease, Austin, Texas, USA*

Parameter	Value				
Incubation period, d (range)	2.9 (1.9–3.9)				
Infectious period, d (range)	6.3 (5.3–7.3)				
Asymptomatic proportion, %	43				
Average hospitalization, d					
Recovered	10.96				
Died	8.2				
Transmission reduction during Stay Home–Work Safe Order, % (95% CI)†	70 (45%–100%)				
Cocooning efficacy, % reduction in transmission relative to Stay–Home Work Safe Order‡					
Cocooning	100				
Enhanced cocooning	125				
Age group, y	0–4	5–17	18–49	50–64	>65
Symptomatic case hospitalization rate, %§					
Low-risk group	0.0279	0.0215	1.3215	2.8563	3.3873
High-risk group	0.2791	0.2146	13.2154	28.5634	33.8733
Infected fatality rate, %‡					
Low-risk group	0.0009	0.0022	0.0339	0.2520	0.6440
High-risk group	0.0092	0.0218	0.3388	2.5197	6.4402

*Detailed parameter distributions and references are given in Appendix Tables 3, 4. †Estimated by fitting the model to coronavirus disease hospitalization counts March 13–April 23. ‡The Appendix provides sensitivity analyses with respect to 2 key assumptions of the model: age-specific contact patterns, which might have changed during the recent unprecedented social distancing; and equally effective cocooning of persons at high risk across all age groups. Cocooning and enhanced cocooning are for persons >65 years of age and persons <65 years of age with high-risk underlying conditions.  §The hospitalization rate and fatality rate for the high-risk group is assumed to be 10 times higher than the corresponding low-risk group in the same age range. The overall hospitalization rate and fatality rate is based on the age-specific values listed in corresponding literature.

**Figure:**
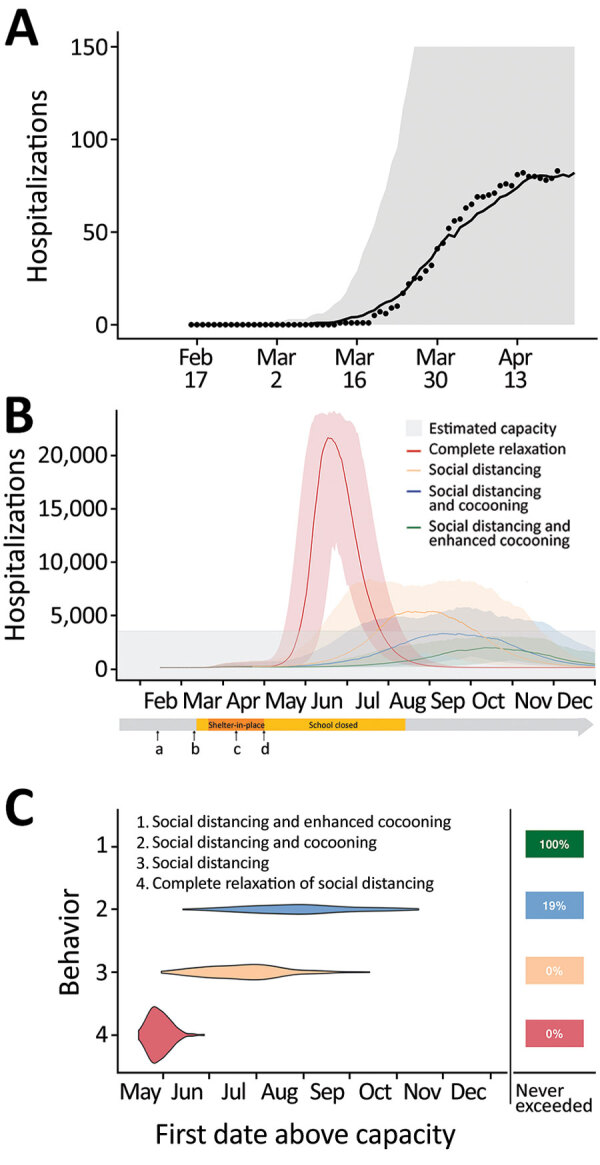
Projected coronavirus disease (COVID-19) hospitalizations during February 16–December 31, 2020, in the Austin-Round Rock Metropolitan Statistical Area, Texas, USA, assuming strict social distancing measures are relaxed on May 1, 2020. A) To calibrate transmission rates before and after Austin’s March 24 Stay Home–Work Safe Order (order 20200324-007; https://www.austintexas.gov), we used least squares to fit our age- and risk-structured susceptible-exposed-infection-recover (SEIR) compartmental model of COVID-19 transmission. Black dots represent daily hospitalization data for the metropolitan area from February 16–April 20, 2020. The curve is the median projection across 200 simulations. Shading represents 95% prediction interval, based on the estimated transmission reduction of 70% beginning March 24. B) Model fitting indicating the ongoing COVID-19 epidemic in Austin. Schools were closed on March 15 and the shelter-in-place order was issued on March 24. a) Date of possible local COVID-19 introduction, February 16; b) date of the first detected case reported, March 13; c) date shelter-in-place order was amended to include cloth face coverings in public, April 13; d) date Texas governor mandated for statewide reopening, May 1. After May 1, we project 4 scenarios in which transmission in low-risk and high-risk groups change relative the reductions achieved during the March 24–May 1 stay-home period: 1) a complete relaxation of measures with transmission rates rebounding to baseline (red); partially relaxed social distancing measures that are 75% as effective as the stay-home order in low-risk groups, with either 2) identical relaxation in high-risk populations (yellow), 3) cocooning that continues to reduce transmission in high-risk groups at the level achieved during the stay-home order (blue), or 4) enhanced cocooning that reduces transmission in high-risk groups further, by 125% relative to the stay home order (green). Lines indicate the median and shading indicates 95% CI across 200 stochastic simulations. Gray shading at bottom indicates 80% of the estimated total daily hospital capacity in the Austin–Round Rock MSA for COVID-19 patients of the 4,299 total beds (3,440). The projections assume that schools open on August 18th. C) The projected first date in 2020 that COVID-19 hospital bed requirements will exceed local capacity for each scenario, as indicated by corresponding colors. The right column indicates the chance that hospitalizations will not exceed capacity in 2020. For example, under enhanced cocooning, we would not expect hospitalizations to exceed capacity.

Cities likely will experience additional waves of COVID-19 when social distancing orders are relaxed. Our model indicates that Austin must aggressively reduce SARS-CoV-2 spread to avoid overwhelming hospital capacity by the end of 2020. Without cocooning, measures that reduce transmission with >90% the efficacy of the stay-home order are needed; with cocooning, social distancing measures for persons at lower risk can be more relaxed (Appendix Figure 1). Cocooning of older adults and persons with known high-risk conditions (*8*) can protect thousands in Austin and millions worldwide. The high-risk population in Austin, as in many cities, is diverse; 66% are >65 years of age, »5,000 are residents in LTCs, and almost 3,000 are persons experiencing homelessness (*9*). Cocooning should be resourced proactively and tailored to meet the distinct needs of high-risk subgroups, including work-at-home and paid leave programs that enable high-risk workers to self-isolate (*10*). Concerted efforts also are needed to shelter residents of LTCs (*3*) and persons experiencing homelessness, where risks are compounded by group living conditions that amplify COVID-19 transmission. Thus, cocooning should be added to the national gating criteria prior to relaxation of social distancing.

Appendix Additional information and modeling parameters for estimating the effects of cocooning on coronavirus disease rates.
