# Supplementary material for: Effects of Cocooning on Coronavirus Disease Rates after Relaxing Social Distancing
Source: Emerg Infect Dis. 2020 Dec;26(12):3066–8. doi: 10.3201/eid2612.201930 (PMC7706923; doi:10.3201/eid2612.201930)
Supplement: Appendix — Additional information and modeling parameters for estimating the effects of cocooning on coronavirus disease rates. [file 20-1930-Techapp-s1.pdf]

Article DOI: https://doi.org/10.3201/eid2612.201930

Effects of Cocooning on Coronavirus Disease Rates after Relaxing Social Distancing

Appendix

Section 1. Appendix Table 1 and Appendix Figure 1

Section 2. Stochastic Compartmental Model of COVID-19 Transmission in the Austin-Round Rock Metropolitan Statistical Area

The model structure is diagrammed in Appendix Figure 2 and described in the equations below.

For each age and risk group, we built a separate set of compartments to model the transitions between the disease states: susceptible (S), exposed (E), symptomatic infectious (I^Y), asymptomatic infectious (I^A), symptomatic infectious that are hospitalized (I^H), recovered (R), and deceased (D). The symbols S, E, I^Y, I^A, I^H, R, and D denote the number of persons in that state in the given age/risk group and the total size of the age/risk group is $N = S + E + I^Y + I^A + I^H + R + D$.

The model for persons in age group a and risk group r is given by:

$$\frac{dS_{a,r}}{dt} = -\sum_{i\in A}\sum_{j\in K}\left(I_{i,j}^{Y}\omega^{Y} + I_{i,j}^{A}\omega^{A} + E_{i,j}\omega^{E}\right)\beta\phi_{a,i}/N_i$$

$$\frac{dE_{a,r}}{dt} = \sum_{i\in A}\sum_{j\in K}\left(I_{i,j}^{Y}\omega^{Y} + I_{i,j}^{A}\omega^{A} + E_{i,j}\omega^{E}\right)\beta\phi_{a,i}/N_i - \sigma E_{a,r}$$

$$\frac{dI_{a,r}^{A}}{dt} = (1-\tau)\sigma E_{a,r} - \gamma^{A} I_{a,r}^{A}$$

$$\frac{dI_{a,r}^{Y}}{dt} = \tau\sigma E_{a,r} - (1-\pi)\gamma^{Y} I_{a,r}^{Y} - \pi\eta I_{a,r}^{Y}$$

$$\frac{dI_{a,r}^H}{dt} = \pi\eta I_{a,r}^Y - (1-\nu)\gamma^H I_{a,r}^H - \nu\mu I_{a,r}^H$$

$$\frac{dR_{a,r}}{dt} = \gamma^A I_{a,r}^A + (1-\pi)\gamma^Y I_{a,r}^Y + (1-\nu)\gamma^H I_{a,r}^H$$

$$\frac{dD_{a,r}}{dt} = \nu\mu I_{a,r}^H$$

where A and K are all possible age and risk groups, ω^A, ω^Y, and ω^H are relative infectiousness of the I^A, I^Y, and E compartments, respectively, β is transmission rate, $\phi_{a,i}$ is the mixing rate between age group a, $i \in A$, γ^A, γ^Y, and γ^H are the recovery rates for the I^A, I^Y, and I^H compartments, respectively, σ is the exposed rate, τ is the symptomatic ratio, π is the proportion of symptomatic persons requiring hospitalization, η is the rate at which hospitalized cases enter the hospital following symptom onset, ν is mortality rate for hospitalized cases, and μ is rate at which terminal patients die.

We model stochastic transitions between compartments by using the τ-leap method (*1*,*2*) with key parameters given in Appendix Table 1. Assuming that the events at each time-step are independent and do not affect the underlying transition rates, the numbers of each type of event should follow Poisson distributions with means equal to the rate parameters. We thus simulate the model according to the following equations:

$S_{a,r}(t+1) - S_{a,r}(t) = -P_1$

$E_{a,r}(t+1) - E_{a,r}(t) = P_1 - P_2$

$I_a^A,r\ (t+1) - I_a^A,r\ (t) = (1-\tau)P\ 2 - P\ 3$

$I_a^Y,r\ (t+1) - I_a^Y,r\ (t) = \tau P\ 2 - P\ 4 - P\ 5$

$I_a^H,r\ (t+1) - I_a^H,r\ (t) = P\ 5 - P\ 6 - P\ 7$

$R_{a,r}(t+1) - R_{a,r}(t) = P_3 + P_4 + P_6$

$D_{a,r}(t+1) - D_{a,r}(t) = P_7$,

with

$P_1 \sim Pois(S_{a,r}(t) F_{a,r}(t))$

$P_2 \sim Pois(\sigma E_{a,r}(t))$

$P_3 \sim Pois(\gamma^A I^A_{a,r}(t))$

$P_4 \sim Pois((1-\pi)\gamma^Y I^Y_{a,r}(t))$

$P_5 \sim Pois(\pi\eta I^Y_{a,r}(t))$

$P_6 \sim Pois((1-\nu)\gamma^H I_a^H)$

$P_7 \sim Pois(\nu\mu I^H_{a,r}(t))$

and where $F_{a,r}$ denotes the force of infection for persons in age group a and risk group r and is given by:

$$F_{a,r}(\mathrm{t}) = \sum_{i\in A}\sum_{j\in K}\left(I^Y_{i,j}(t)\omega^Y + I^A_{i,j}(t)\omega^A + E_{i,j}(t)\omega^E\right)\beta_{a,i}\,\phi_{a,i}/N_i.$$

Parameter Estimation using Austin Hospitalization Data

The city of Austin provided the total number of *heads in beds* for confirmed COVID-19 patients in hospitals in Austin-Round Rock MSA from March 13 to April 24, 2020 (Appendix Table 2). Let $H(t)$ be the observed and $\hat{H}(t)$ be the predicted hospitalization totals on day t, where predictions are made from the deterministic model formulation. We conducted least-squares fitting to estimate β,κ,t_0, corresponding to the baseline transmission rate, the reduction in contacts following Austin's Stay Home–Work Safe Order, and the initial seed date of the epidemic respectively.

Fitting was conducted by using the nonlinear least squares method made available in SciPy (*3*), which minimizes the least squares error defined as $LSE = (H(t) - \hat{H}(t))^2$ (*3*). The best fit model accurately captured the hospitalization data and estimated $\hat{\beta} = 0.035$, $\hat{\kappa} = 0.95$, $\hat{t}_0 =$ February 16, 2020.

We calculated 95% confidence intervals for $\hat{\kappa}$ by comparing prediction intervals from stochastic simulations with the hospitalization data. We ran 500 stochastic simulations for each of the following possible values of κ': 0.0, 0.05, ..., 0.95, 1.0. For each value of κ', we conducted the following analysis to determine if κ' lies inside the 95% confidence interval for κ'.

- For all simulations, we calculate the day-to-day difference in hospitalizations (i.e., *heads in beds*) during the period following the Stay Home–Work Safe order: $\hat{z}_t = \hat{H}_t - \hat{H}_{t-1}$. We do the same for the actual data: $z_t = \overline{H_t} - \overline{H_{t-1}}$.

- We compute the 95% prediction interval for $\hat{z}_t$ across all 500 stochastic simulations for κ′ for each day *t*.
- We then conduct a test of the null hypothesis H_0: $\kappa' = \kappa$. Under this null hypothesis, we would expect roughly 95% of the observed data (z_t) to fall within the 95% prediction band for $\hat{z}_t$ that we constructed from our simulations. By analyzing the day-to-day difference in hospitalizations rather than daily hospitalizations, we can assume that the data are independent from one day to the next. Then the expected number of observed values contained in the 95% prediction band is given by the binomial expression:

$$N_{observed} \sim B\,(N_{points}, 0.95)$$

where $N_{observed}$ is the number of data points contained within the 95% prediction band and N_{points} is the total number of data points (i.e., days).

- We calculate $N_{contained}$, the actual number of data points contained within the 95% prediction band, and compute a p-value by identifying the probability that one would observe $N_{contained}$ or more extreme results under the null distribution. If $p<0.05$, we reject the null hypothesis H_0: $\kappa' = \kappa$.

Model Parameters

Model parameters are provided in Appendix Tables 3–9.

Section 3. Sensitivity Analyses

Sensitivity Analysis with Respect to Age-Specific Contact Rates

We conducted a sensitivity analysis in which we modeled the same 4 scenarios but without any age-specific contact rates. That is, we removed the contact matrices altogether and assume that transmission rates are homogeneous across the population. Under these conditions, we would expect cocooning to have an even larger beneficial effect (Appendix Figure 3). Specifically, 9% of the 200 simulations exceed hospital capacity with cocooning assuming homogeneous contact rates, where the number is 19% with contact matrices. The reduction in peak hospitalization with cocooning is also higher when assuming homogeneous mixing. This likely stems from our primary model (with contact matrices) assuming that persons ≥65 years of

age have fewer contacts on average than younger adults and children. In a sense, they are naturally cocooned by their baseline behavior. In the homogeneous contact model, this large high-risk group is more exposed, and thus even moderate cocooning has a large protective effect.

Sensitivity Analysis with Respect to Cocooning of High-Risk Persons <65 Years of Age

In the cocooned population, 34% are ≥65 years of age and 66% are younger persons with ≥1 chronic condition, as described in Appendix Section 4. When we restrict cocooning in our model to protect only persons ≥65 years of age, the projected epidemiologic effects are reduced (Appendix Figure 4). Not only does this reduce protection to only 34% of the vulnerable population, but it targets the subset of the high-risk population with the lowest contact rates. The younger high-risk populations who remain exposed are more likely to become infected and infect others because of their higher rates of daily contacts.

Section 4. Estimation of Age-Stratified Proportion of Population at High Risk for COVID-19 Complications

We estimated age-specific proportions of the population at high risk for complications from COVID-19 based on data for Austin, TX and Round-Rock, TX from the 500 Cities Project by the US Centers for Disease Control and Prevention (CDC) (*16*; Appendix Figure 5).

We assumed that high-risk conditions for COVID-19 are the same as those specified for influenza by the CDC (*10*). The CDC’s 500 Cities Project provides city-specific estimates of prevalence for several of these conditions among adults (*23*). The estimates were obtained from the 2015–2016 Behavioral Risk Factor Surveillance System (BRFSS, https://www.cdc.gov/brfss/index.html) data by using a small-area estimation methodology called multilevel regression and poststratification (*11*,*12*), which links geocoded health surveys to high spatial resolution population demographic and socioeconomic data (*12*).

Estimating High-Risk Proportions for Adults

To estimate the proportion of adults at high risk for complications, we used CDC’s 500 cities data, as well as data on the prevalence of HIV/AIDS, obesity, and pregnancy among adults (Appendix Table 10).

The CDC 500 cities dataset includes the prevalence of each condition on its own, rather than the prevalence of multiple conditions (e.g., dyads or triads). Thus, we use separate

comorbidity estimates to determine overlap. Reference about chronic conditions (*24*) gives United States estimates for the proportion of the adult population with 0, 1, or $\geq$2 chronic conditions, per age group. By using this and the 500 cities data we can estimate the proportion of the population (pHR) in each age group in each city with $\geq$1 chronic condition listed in the CDC 500 cities data (Appendix Table 10) putting them at high-risk for complications from influenza.

HIV

We used the data from Table 20a in CDC HIV surveillance report (*17*) to estimate the population in each risk group living with HIV in the United States (last column, 2015 data). Assuming independence between HIV and other chronic conditions, we increased the proportion of the population at high risk for influenza to account for persons with HIV but no other underlying conditions.

Morbid Obesity

A body mass index (BMI) >40 kg/m^2 indicates morbid obesity and is considered high risk for influenza. The 500 Cities Project reports the prevalence of obese persons in each city with BMI >30 kg/m^2 (not necessarily morbid obesity). We use the data from Table 1 in Sturm and Hattori (*18*) to estimate the proportion of persons with BMI >30 kg/m^2 that actually have BMI >40 kg/m^2 across the United States; we then apply this to the 500 cities obesity data to estimate the proportion of persons who are morbidly obese in each city. Table 1 of Morgan et al. (*19*) suggests that 51.2% of morbidly obese adults have $\geq$1 other high-risk chronic condition, and update our high-risk population estimates accordingly to account for overlap.

Pregnancy

We separately estimated the number of pregnant women in each age group and each city, following the methodology in CDC reproductive health report (*25*). We assume independence between any of the high-risk factors and pregnancy and further assume that half the population are women.

Estimating High-Risk Proportions for Children

Since the 500 Cities Project only reports data for adults $\geq$18 years of age, we took a different approach to estimating the proportion of children at high risk for severe influenza. The 2 most prevalent risk factors for children are asthma and obesity; we also accounted for childhood diabetes, HIV, and cancer.

From Miller et al. (*26*), we obtained national estimates of chronic conditions in children. For asthma, we assumed that variation among cities would be similar for children and adults. Thus, we used the relative prevalence of asthma in adults to scale our estimates for children in each city. The prevalence of HIV and cancer in children are taken from CDC HIV surveillance report (*18*) and cancer research report (*27*).

We first estimated the proportion of children having either asthma, diabetes, cancer, or HIV, assuming no overlap in these conditions. We estimated city-level morbid obesity in children by using the estimated morbid obesity in adults multiplied by a national constant ratio for each age group estimated from Hales et al. (*28*) that represents the prevalence in morbid obesity in children given the prevalence observed in adults. From Morgan et al. (*19*), we estimated that 25% of morbidly obese children have another high-risk condition and adjusted our final estimates accordingly.

Resulting Estimates

We compared our estimates for the Austin-Round Rock MSA to published national-level estimates (*29*) of the proportion of each age group with underlying high-risk conditions (Appendix Table 11). The biggest difference was observed in older adults, with Austin having a lower proportion at risk for complications from COVID-19 than the national average; for persons 25–39 years of age, the high-risk proportion was slightly higher than the national average (Appendix Figure 5).

References

1. Keeling MJ, Rohani P. Modeling infectious diseases in humans and animals. Princeton (MA): Princeton University Press; 2011.

2. Gillespie DT. Approximate accelerated stochastic simulation of chemically reacting systems. J Chem Phys. 2001;115:1716–33. https://doi.org/10.1063/1.1378322

3. The SciPy Community. scipy.optimize.least_squares—SciPy v1.4.1 reference guide. [cited 2020 Apr 26]. https://docs.scipy.org/doc/scipy/reference/generated/scipy.optimize.least_squares.html

4. Prem K, Cook AR, Jit M. Projecting social contact matrices in 152 countries using contact surveys and demographic data. PLOS Comput Biol. 2017;13:e1005697. PubMed https://doi.org/10.1371/journal.pcbi.1005697

5. Mossong J, Hens N, Jit M, Beutels P, Auranen K, Mikolajczyk R, et al. Social contacts and mixing patterns relevant to the spread of infectious diseases. PLoS Med. 2008;5:e74. PubMed https://doi.org/10.1371/journal.pmed.0050074

6. He X, Lau EHY, Wu P, Deng X, Wang J, Hao X, et al. Temporal dynamics in viral shedding and transmissibility of COVID-19. Nat Med. 2020;26:672–5 PubMed. https://doi.org/10.1038/s41591-020-0869-5

7. Gudbjartsson DF, Helgason A, Jonsson H, Magnusson OT, Melsted P, Norddahl GL, et al. Spread of SARS-CoV-2 in the Icelandic Population. N Engl J Med. 2020;382:2302–15. PubMed https://doi.org/10.1056/NEJMoa2006100

8. Zhang J, Litvinova M, Wang W, Wang Y, Deng X, Chen X, et al. Evolving epidemiology and transmission dynamics of coronavirus disease 2019 outside Hubei province, China: a descriptive and modelling study. Lancet Infect Dis. 2020;20:793–802. PubMed https://doi.org/10.1016/S1473-3099(20)30230-9

9. Verity R, Okell LC, Dorigatti I, Winskill P, Whittaker C, Imai N, et al. Estimates of the severity of coronavirus disease 2019: a model-based analysis. Lancet Infect Dis. 2020;20:669–77. PubMed https://doi.org/10.1016/S1473-3099(20)30243-7

10. US Centers for Disease Control and Prevention. People at high risk for flu complications. 2019 [cited 2020 Mar 26]. https://www.cdc.gov/flu/highrisk

11. US Centers for Disease Control and Prevention. Behavioral Risk Factor Surveillance System (BRFSS). 2019 [cited 2020 Jun 22]. https://www.cdc.gov/brfss

12. Zhang X, Holt JB, Lu H, Wheaton AG, Ford ES, Greenlund KJ, et al. Multilevel regression and poststratification for small-area estimation of population health outcomes: a case study of chronic obstructive pulmonary disease prevalence using the behavioral risk factor surveillance system. Am J Epidemiol. 2014;179:1025–33. PubMed https://doi.org/10.1093/aje/kwu018

13. Stokes EK, Zambrano LD, Anderson KN, Marder EP, Raz KM, El Burai Felix S, et al. Coronavirus disease 2019 case surveillance—United States, January 22–May 30, 2020. MMWR Morb Mortal Wkly Rep. 2020;69:759–65. PubMed https://doi.org/10.15585/mmwr.mm6924e2

14. Austin Independent School District. Calendar of events [cited 2020 Mar 26]. https://www.austinisd.org/calendar

15. Tindale L, Coombe M, Stockdale JE, Garlock E, Lau WYV, Saraswat M, et al. Transmission interval estimates suggest pre-symptomatic spread of COVID-19. eLife. 2020;9:e57149. PubMed https://doi.org/10.7554/eLife.57149

16. US Centers for Disease Control and Prevention. 500 Cities project: local data for better health. 2019 [cited 2020 Jun 22]. https://www.cdc.gov/500cities

17. US Centers for Disease Control and Prevention. HIV surveillance report, 2016 [cited 2020 Jun 1]. http://www.cdc.gov/hiv/library/reports/hiv-surveillance.html

18. Sturm R, Hattori A. Morbid obesity rates continue to rise rapidly in the United States. Int J Obes. 2013;37:889–91. PubMed https://doi.org/10.1038/ijo.2012.159

19. Morgan OW, Bramley A, Fowlkes A, Freedman DS, Taylor TH, Gargiullo P, et al. Morbid obesity as a risk factor for hospitalization and death due to 2009 pandemic influenza A(H1N1) disease. PLoS One. 2010;5:e9694. PubMed https://doi.org/10.1371/journal.pone.0009694

20. Martin JA, Hamilton BE, Osterman MJK, Driscoll AK, Drake P. Births: final data for 2017. Natl Vital Stat Rep. 2018;67:1–50 PubMed

21. Jatlaoui TC, Boutot ME, Mandel MG, Whiteman MK, Ti A, Petersen E, et al. Abortion surveillance—United States, 2015. MMWR Surveill Summ. 2018;67:1–45. PubMed https://doi.org/10.15585/mmwr.ss6713a1

22. Ventura SJ, Curtin SC, Abma JC, Henshaw SK. Estimated pregnancy rates and rates of pregnancy outcomes for the United States, 1990–2008. Natl Vital Stat Rep. 2012;60:1–21. PubMed

23. US Centers for Disease Control and Prevention. 500 Cities project: health outcomes. 2019 [cited 2020 Jun 22]. https://www.cdc.gov/500cities/definitions/health-outcomes.htm

24. Fox S, Duggan M. Pew Research Center. The diagnosis difference. Part one: who lives with chronic conditions. 2013 [cited 2020 Jun 22]. https://www.pewresearch.org/internet/2013/11/26/part-one-who-lives-with-chronic-conditions

25. US Centers for Disease Control and Prevention. Estimating the number of pregnant women in a geographic area [cited 2020 Jun 1]. https://www.cdc.gov/reproductivehealth/emergency/pdfs/PregnacyEstimatoBrochure508.pdf

26. Miller GF, Coffield E, Leroy Z, Wallin R. Prevalence and costs of five chronic conditions in children. J Sch Nurs. 2016;32:357–64. PubMed https://doi.org/10.1177/1059840516641190

27. American Cancer Society. Cancer facts and figures 2014 [cited 2020 Jun 22]. https://www.cancer.org/research/cancer-facts-statistics/all-cancer-facts-figures/cancer-facts-figures-2014.html

28. Hales CM, Fryar CD, Carroll MD, Freedman DS, Ogden CL. Trends in obesity and severe obesity prevalence in US youth and adults by sex and age, 2007–2008 to 2015–2016. JAMA. 2018;319:1723–5. PubMed https://doi.org/10.1001/jama.2018.3060

29. Zimmerman RK, Lauderdale DS, Tan SM, Wagener DK. Prevalence of high-risk indications for influenza vaccine varies by age, race, and income. Vaccine. 2010;28:6470–7. PubMed https://doi.org/10.1016/j.vaccine.2010.07.037

Appendix Table 1. Estimated time until coronavirus disease hospitalizations exceed local hospital bed surge capacity during February 16–December 31, 2020 based on effectiveness of various public health measures, Austin-Round Rock metropolitan statistical area, Texas, USA*

	% Effectiveness†				
Measures	0	50	75	90	95
No cocooning					
No. days to exceed surge capacity	27 (16–43)	44 (26–77)	84 (41–137)	NE (133–NE)	NE
Cumulative no. hospital beds	82,146 (79,331–84,276)	68,403 (62,593–72,733)	52,452 (45,748–59,083)	31,018 (5,332–41,805)	14,247 (505–33,329)
Cumulative no. deaths	10,139 (9,598–10,509)	8,046 (7,143–8,703)	5,822 (4,939–6,791)	3,192 (437–4,501)	1,340 (44–3,510)
Cocooning					
No. days to exceed surge capacity	38 (25–58)	62 (38–114)	127 (63–NE)	NE	NE
Cumulative no. hospital beds	49,637 (45,173–53,119)	44,447 (38,490–49,568)	37,449 (27,611–43,685)	21,411 (1,625–33,957)	9,905 (336–27,609)
Cumulative no. deaths	5,156 (4,535–5,611)	4,622 (3,886–5,290)	3,857 (2,714–4,618)	2,076 (141–3,505)	894 (32–2,740)
Enhanced cocooning					
Days to exceed surge capacity	47 (34–68)	87 (57–173)	NE	NE	NE
Cumulative hospital beds	30,778 (28,133–33,544)	26,407 (23,355–29,384)	19,927 (10,070–24,641)	3,371 (35–13,771)	838 (56–7,075)
Cumulative deaths	2,745 (2,473–3,048)	2,362 (2,056–2,698)	1,755 (770–2,276)	260 (2–1,187)	77 (5–613)

*Assuming that social distancing measures are relaxed on May 1, 2020. Values are expressed as median (95% CI) across 200 stochastic simulations based on the parameters given in Appendix. NE, not expected to surpass the specified thresholds before December 31, 2020.
†Compared with Stay Home–Work Safe order.

Appendix Table 2. Number of persons hospitalized for coronavirus disease each day during March 13–April 24, 2020, Austin-Round Rock metropolitan area, Texas, USA

Date	No. persons hospitalized
13 Mar 2020	1
14 Mar 2020	1
15 Mar 2020	1
16 Mar 2020	1
17 Mar 2020	1
18 Mar 2020	1
19 Mar 2020	5
20 Mar 2020	7
21 Mar 2020	6
22 Mar 2020	9
23 Mar 2020	10
24 Mar 2020	17
25 Mar 2020	22
26 Mar 2020	25
27 Mar 2020	25
28 Mar 2020	29
29 Mar 2020	32
30 Mar 2020	41
31 Mar 2020	44
1 Apr 2020	52
2 Apr 2020	56
3 Apr 2020	57
4 Apr 2020	63
5 Apr 2020	65
6 Apr 2020	69
7 Apr 2020	69
8 Apr 2020	70
9 Apr 2020	71
10 Apr 2020	75
11 Apr 2020	76
12 Apr 2020	75
13 Apr 2020	81
14 Apr 2020	82
15 Apr 2020	80
16 Apr 2020	80
17 Apr 2020	79
18 Apr 2020	78
19 Apr 2020	79
20 Apr 2020	83
21 Apr 2020	78
22 Apr 2020	82
23 Apr 2020	78
24 Apr 2020	75

Appendix Table 3. Initial conditions, school closures, and social distancing policies for coronavirus disease during 2020 in the Austin-Round Rock metropolitan statistical area, Texas, USA

Variable	Settings
Initial day of simulation	16 Feb 2020
Initial infection number in locations	1 symptomatic case in 18–49 y age group
School closures	15 Mar–17 Aug 2020
Age-specific and day-specific contact rates*	Home, work, other and school matrices provided in Appendix Tables 6–9
During 16 Feb–18 Mar	
Weekday	Home + school + work + other
Weekend	Home + other
Weekday holiday	Home + other
During 19–24 Mar	
Weekday	Home + work + other
Weekend	Home + other
Weekday holiday	Home + other
During 25 Mar–17 Aug	
Weekday	(1 – κ) × (home + work + other)
Weekend	(1 – κ) × (home + other)
Weekday holiday	(1 – κ) × (home + other)
During 18 Aug–Dec 31	
Weekday	(1 – κ) × (home + school + work + other)
Weekend	(1 – κ) × (home + other)
Weekday holiday	(1 – κ) × (home + other)

*We assume the age-specific contact rates given in (*4*), which takes the contact numbers estimated through diary-based POLYMOD study in Europe (*5*) and extrapolates to the United States. The values in Appendix Tables 6–9 are the assumed daily contacts between each pair of age groups at home, school, work, and all other places, respectively. For example, the value of 2.0 in Table A6 row 1 column 2 means that 1 person in the 0–4 age group is estimated to contact 2 people daily in the 18–64 age group at home. These contact matrices are used to adjust the transmission rate between age groups. The accuracy of the contact matrices is limited by the following: possible biases with the original diary-based study (*5*); assumptions made when projecting the original study to the United States (*4*); and impacts of coronavirus disease policies and perceptions on daily contact patterns.

Appendix Table 4. Model parameters*

Parameters	Values	Source
R_0, basic reproduction number	2.8	Derived from fitted model
δ, doubling time before intervention, d	2.9	Derived from fitted model
β, baseline transmission rate	0.057	Fitted to daily COVID-19 hospitalizations in Austin-Round Rock MSA, Texas
κ, reduction in transmission		During 25 Mar–1 May 2020, fitted to daily COVID-19 hospitalizations in Austin-Round Rock MSA, Texas
15 Feb–24 Mar 2020	0	
25 Mar–1 May 2020	0.7 (95% CI 0.7–1)	
25 Mar–31 Dec 2020		
Scenario 1	0	
Scenario 2	0.5	
Scenario 3	0.7	
Scenario 4	0.9	
Scenario 5	0.95	
c, cocooning efficacy; the reduction in transmission relative to Austin's Stay Home–Work Safe Order for all high-risk groups		Assumption
Cocooning	1.0	
Enhanced cocooning	1.25	
γ^A, recovery rate on asymptomatic compartment	Equal to γ^Y	
γ^Y, recovery rate on symptomatic nontreated compartment	$\frac{1}{\gamma^Y} \sim$ *Triangular* (5.3, 6.3, 7.3) $\approx\gamma$	(*6*)
τ, symptomatic proportion, %	57	(*7*)
σ, exposed rate†	$\frac{1}{\sigma} \sim$ *Triangular* (1.9, 2.9, 3.9) $\approx\sigma$	(*6*,*8*,)
ω^A, relative infectiousness of infectious persons in compartment I^A	0.67	(*6*)
IFR, infected fatality ratio, age specific, %		Age adjusted from R. Verity et al. (*9*)
Low-risk group		
0–4 y	0.0009	
5–17 y	0.0022	
18–49 y	0.0339	
50–64 y	0.2520	

Parameters	Values	Source
≥65 y	0.6440	
High-risk group		
0–4 y	0.0092	
5–17 y	0.0218	
18–49 y	0.3388	
50–64 y	2.5197	
≥65 y	6.4402	
YFR, symptomatic fatality ratio, age-specific, %		$Y FR = \underline{IF}\tau\underline{R}$
Low-risk group		
0–4 y	0.0011165	
5–17 y	0.0027	
18–49 y	0.0412	
50–64 y	0.3069	
≥65 y	0.7844	
High-risk group		
0–4 y	0.0112	
5–17 y	0.0265	
18–49 y	0.4126	
50–64 y	3.0690	
≥65 y	7.8443	
h, high-risk proportion, age-specific, %		(*10*–*12*)‡
0–4 y	8.2825	
5–17 y	14.1121	
18–49 y	16.5298	
50–64 y	32.9912	
≥65 y	47.0568	
rr, relative risk for high-risk compared with low-risk persons in an age group	10	(*13*)
School calendars	Austin Independent School District calendar (2019–20, 2020–21)	(*14*)

*CDC, US Centers for Disease Control and Prevention; COVID-19, coronavirus disease; MSA, metropolitan statistical area.
†Based on incubation (*8*) and presymptomatic periods (*6*).
‡Estimated using 2015–2016 Behavioral Risk Factor Surveillance System (BRFSS; https://www.cdc.gov/brfss/index.html) data with multilevel regression and poststratification by using US Centers for Disease Control and Prevention's list of conditions that may increase the risk for serious complications from influenza.

Appendix Table 5. Hospitalization parameters

Parameters	Values	Source
γ^H: recovery rate in hospitalized compartment	1/14	14 d-average from admission to discharge (UT Austin Dell Med)
YHR: symptomatic case hospitalization rate (%)		Age adjusted from R. Verity et al. (*9*)
Low-risk group		
0–4 y	0.0279	
5–17 y	0.0215	
18–49 y	1.3215	
50–64 y	2.8563	
≥65 y	3.3873	
High-risk group		
0–4 y	0.2791	
5–17 y	0.2146	
18–49 y	13.2154	
50–64 y	28.5634	
≥65 y	33.8733	
π, rate of symptomatic individuals go to hospital, age-specific	$\pi = \frac{\gamma^Y \times YHR}{\eta + (\gamma^Y - \eta)YHR}$	
η, rate from symptom onset to hospitalized	0.1695	5.9-day average from symptom onset to hospital admission Tindale et al. (*15*)
μ, rate from hospitalized to death	1/14	14-day average from admission to death (UT Austin Dell Med)
HFR, hospitalized fatality ratio, age-specific, %		$HFR = \frac{IFR}{YHR(1-\tau)}$

Parameters	Values	Source
0–4 y	4	$\nu = \frac{\gamma^H HFR}{\mu + (\gamma^H - \mu)HFR}$
5–17 y	12.365	
18–49 y	3.122	
50–64 y	10.745	
≥65 y	23.158	
ν, death rate on hospitalized persons, age-specific		
0–4 y	0.0390	
5–17 y	0.1208	
18–49 y	0.0304	
50–64 y	0.1049	
≥65 y	0.2269	
Healthcare capacity, no. hospital beds	4,299	Estimates provided by each of the region's hospital systems and aggregated by regional public health leaders

Appendix Table 6. Home contact matrix (daily number contacts by age group at home)

Age, y	0–4	5–17	18–49	50–64	≥65
0–4	0.5	0.9	2.0	0.1	0.0
5–17	0.2	1.7	1.9	0.2	0.0
18–49	0.2	0.9	1.7	0.2	0.0
50–64	0.2	0.7	1.2	1.0	0.1
≥65	0.1	0.7	1.0	0.3	0.6

Appendix Table 7. School contact matrix (daily number contacts by age group at school)

Age, y	0–4	5–17	18–49	50–64	≥65
0–4	1.0	0.5	0.4	0.1	0.0
5–17	0.2	3.7	0.9	0.1	0.0
18–49	0.0	0.7	0.8	0.0	0.0
50–64	0.1	0.8	0.5	0.1	0.0
≥65	0.0	0.0	0.1	0.0	0.0

Appendix Table 8. Work contact matrix (daily number contacts by age group at work)

Age, y	0–4	5–17	18–49	50–64	≥65
0–4	0.0	0.0	0.0	0.0	0.0
5–17	0.0	0.1	0.4	0.0	0.0
18–49	0.0	0.2	4.5	0.8	0.0
50–64	0.0	0.1	2.8	0.9	0.0
≥65	0.0	0.0	0.1	0.0	0.0

Appendix Table 9. Others contact matrix (daily number contacts by age group at other locations)

Age, y	0–4	5–17	18–49	50–64	≥65
0–4	0.7	0.7	1.8	0.6	0.3
5–17	0.2	2.6	2.1	0.4	0.2
18–49	0.1	0.7	3.3	0.6	0.2
50–64	0.1	0.3	2.2	1.1	0.4
≥65	0.0	0.2	1.3	0.8	0.6

Appendix Table 10. Underlying conditions that put persons at high risk for influenza and data sources for prevalence estimation used in this model

Condition	Data source
Cancer (except skin), chronic kidney disease, COPD, coronary heart disease, stroke, asthma, diabetes	US Centers for Disease Control and Prevention (CDC) 500 cities (*16*)
HIV/AIDS	CDC HIV Surveillance report (*17*)
Obesity	CDC 500 cities (*16*); Sturm and Hattori (*18*); Morgan et al. (*19*)
Pregnancy	National Vital Statistics Reports (*20*) and abortion data (*21*)

Appendix Table 11. Comparison between published national estimates of the percentage of the population at high risk for complications of influenza or coronavirus disease and the percentage of population of Austin-Round Rock metropolitan statistical, Texas, USA

Age group	National estimates (*27*)	Austin (excluding pregnancy)	Pregnant women (proportion of age group)
0–<6 mo	NA	6.8	–
6 mo–4 y	6.8	7.4	–
5–9 y	11.7	11.6	–
10–14 y	11.7	13.0	–
15–19 y	11.8	13.3	1.7
20–24 y	12.4	10.3	5.1
25–34 y	15.7	13.5	7.8
35–39 y	15.7	17.0	5.1
40–44 y	15.7	17.4	1.2
45–49 y	15.7	17.7	–
50–54 y	30.6	29.6	–
55–59 y	30.6	29.5	–
60–64 y	30.6	29.3	–
65–69 y	47.0	42.2	–
70–74 y	47.0	42.2	–
≥75 y	47.0	42.2	–

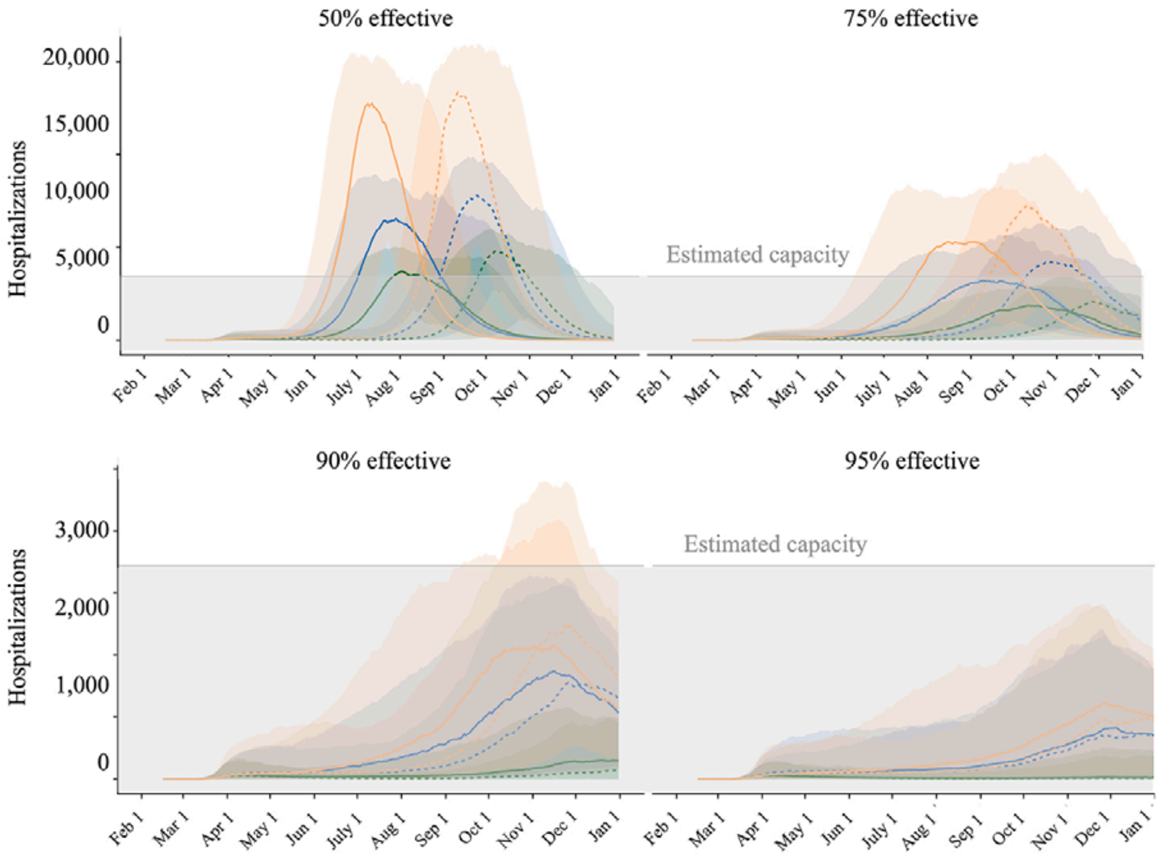

Appendix Figure 1. Projected daily coronavirus disease (COVID-19) hospitalizations during February 16–December 31, 2020 in the Austin-Round Rock metropolitan statistical area with different degrees of transmission reduction after the relaxation of the Stay Home–Work Safe order. Solid lines indicate relaxation of Stay Home–Work Safe order on May 1. Dashed lines indicate relaxation of the order on July 1. Before May 1, we estimated that social distancing reduced COVID-19 transmission by 70% relative to the baseline before school closures in Austin on March 15. After May 1, we considered relaxation of the stay-home orders for low-risk groups as scenarios in which transmission was only 50% (top left), 75% (top right), 90% (bottom left), and 95% (bottom right) as effective as during the Stay Home–Work Safe order. Blue lines assume cocooning of vulnerable populations; that is, everyone ≥65 years of age and persons with high-risk underlying conditions continue to social distance and take precautions that reduce their infection risk the same level as the 70% stay-home order. Green lines assume enhanced cocooning that is 125% as effective as the stay-home order. The yellow lines project COVID-19 cases assuming vulnerable populations have the same transmission reduction as the rest of the population. Lines and shading indicate the median and 95% prediction interval across 200 stochastic simulations.

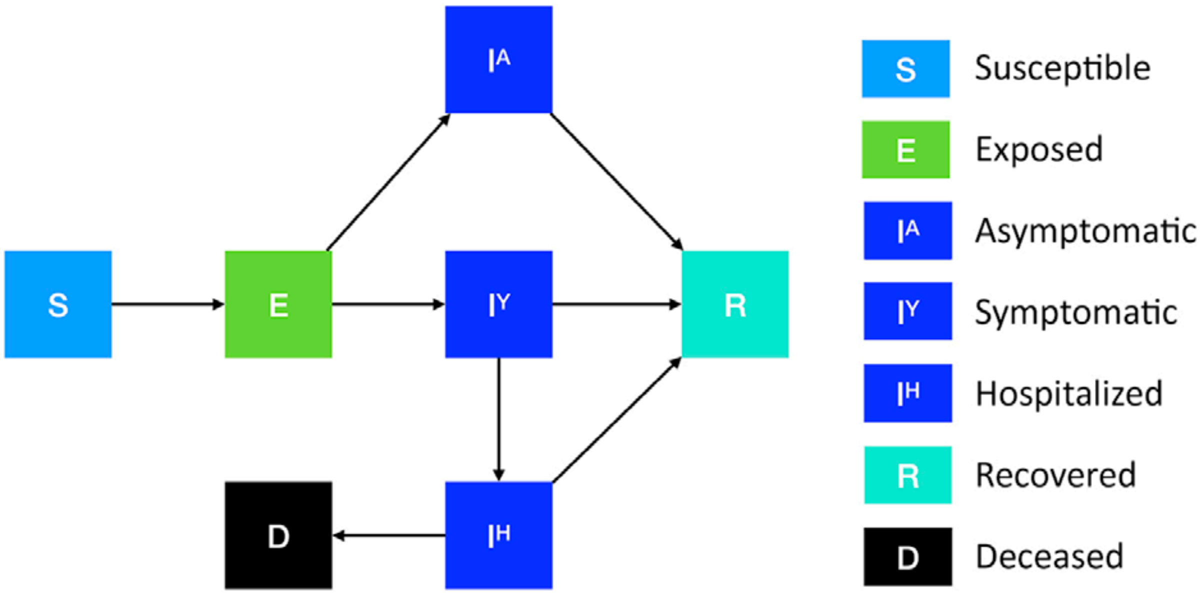

Appendix Figure 2. Compartmental model of coronavirus disease (COVID-190 transmission in a US city. Each subgroup (defined by age and risk) is modeled with a separate set of compartments. Upon infection, susceptible individuals (*S*) progress to exposed (*E*) and then to either symptomatic infectious (I^Y) or asymptomatic infectious (I^A). All asymptomatic cases eventually progress to a recovered class where they remain protected from future infection (*R*); symptomatic cases are either hospitalized (I^H) or recover. Mortality (*D*) varies by age group and risk group and is assumed to be preceded by hospitalization.

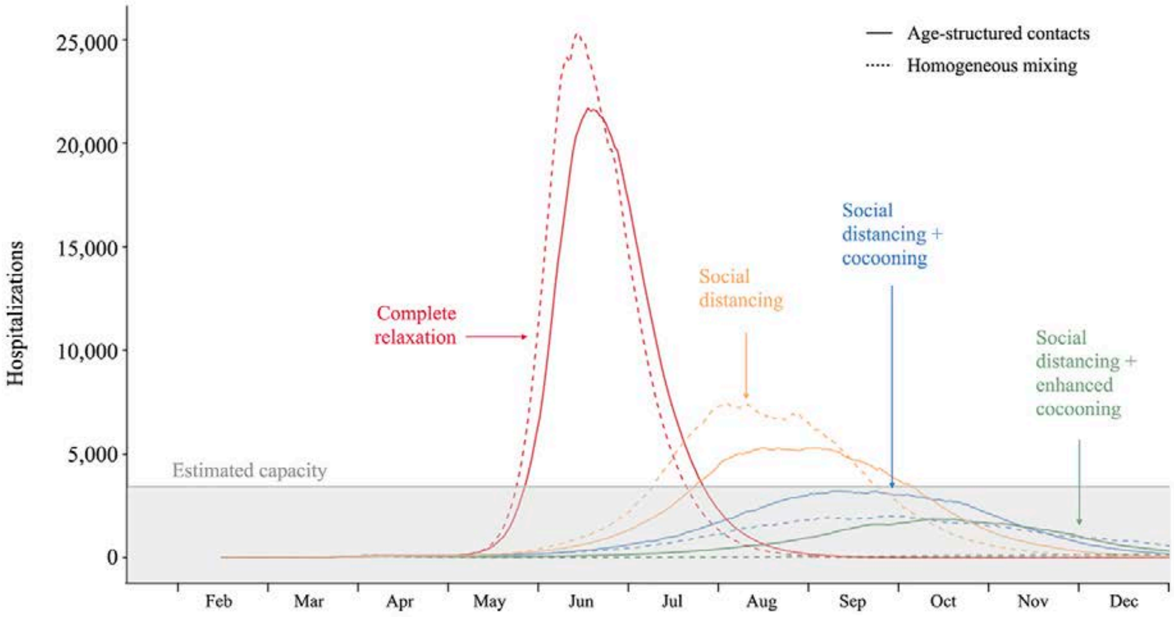

Appendix Figure 3. Sensitivity analysis of hospitalizations in the Austin-Round Rock MSA from February 16 to December 31, 2020 assuming strict social distancing measures are relaxed on May 1, 2020. Solid lines indicate original age-structured contact rates; dashed lines indicate homogeneous mixing. Curves indicate median projections of COVID-19 hospitalizations. The model fitting indicates that the ongoing COVID-19 epidemic in Austin was seeded by a local case around February 16, 2020; the first detected case was reported on March 13, 2020, schools were closed on March 15, and the shelter-in-place order was issued on March 24 and then amended to require cloth face coverings in public on April 13, 2020; the Texas governor mandated statewide reopening beginning May 1. We estimate that transmission was reduced by 70% under the original model (solid) and 75% under the homogeneous model (dashed) beginning March 24th. Following the May 1, we project four scenarios in which transmission in low risk and high risk groups change relative the reductions achieved during the March 24–May 1 stay-home period: (i) a complete relaxation of measures with transmission rates rebounding to baseline (red lines); partially relaxed social distancing measures that are 75% as effective as the stay-home order in low risk groups, with either (ii) identical relaxation in high risk populations (yellow lines), (iii) cocooning that continues to reduce transmission in high risk groups at the level achieved during the stay-home order (blue lines), or (iv) enhanced cocooning that reduces transmission in high risk groups further, by 125% relative to the stay home order (green lines).

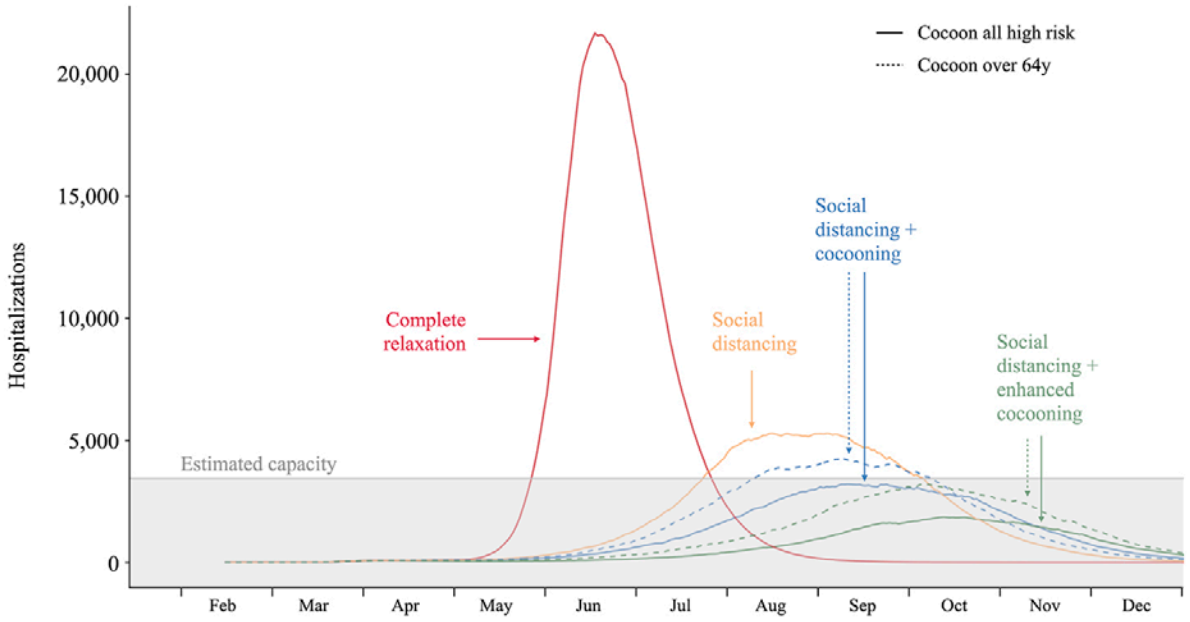

Appendix Figure 4. Sensitivity analysis of hospitalizations in the Austin-Round Rock MSA from February 16–December 31, 2020 assuming strict social distancing measures are relaxed on May 1, 2020. Solid lines indicate cocooning of all high-risk persons; dashed lines indicate cocooning only persons ≥65 years of age. Curves indicate median projections of COVID-19 hospitalizations. The model fitting indicates that the ongoing COVID-19 epidemic in Austin was seeded by a local case around February 16, 2020; the first detected case was reported on March 13, 2020, schools were closed on March 15, and the shelter-in-place order was issued on March 24 and then amended to require cloth face coverings in public on April 13, 2020; the Texas governor mandated statewide reopening beginning May 1. We estimate that transmission was reduced by 70% beginning March 24. Following May 1, we project 4 scenarios in which transmission in low-risk and high-risk groups changes relative to reductions achieved during the March 24–May 1 stay-home period: (i) a complete relaxation of measures with transmission rates rebounding to baseline (red lines); partially relaxed social distancing measures that are 75% as effective as the stay-home order in low risk groups, with either (ii) identical relaxation in high risk populations (yellow lines) or (iii) cocooning that continues to reduce transmission in high risk groups at the level achieved during the stay-home order (blue lines); or (iv) enhanced cocooning that reduces transmission in high risk groups further, by 125% relative to the stay home order (green lines).

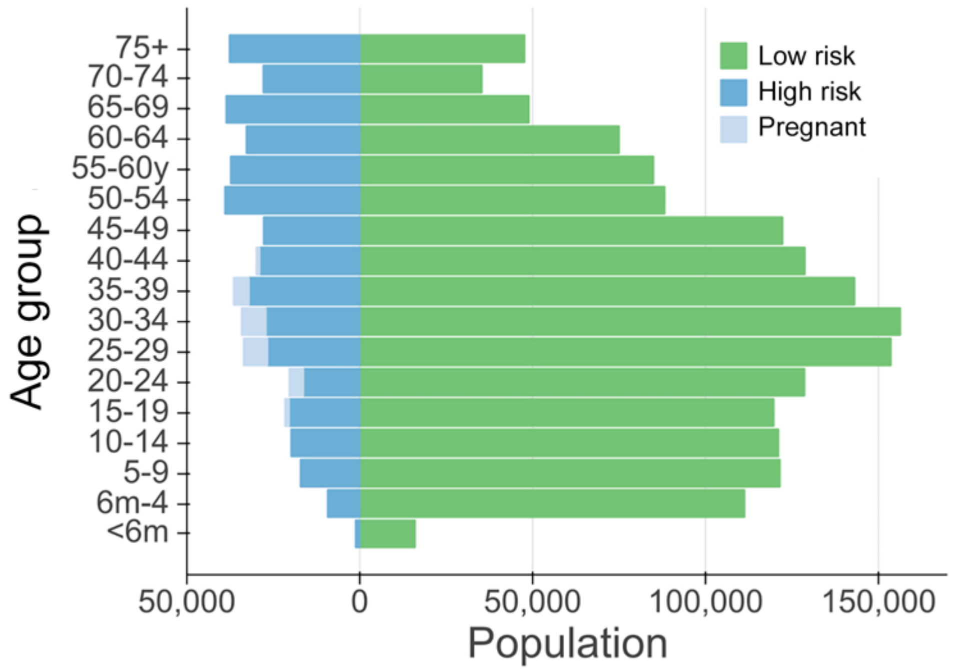

Appendix Figure 5. Demographic and risk composition of the Austin-Round Rock population. Bars indicate age-specific population sizes, separated by low-risk, high-risk, and pregnant persons. High-risk persons are defined as persons with cancer, chronic kidney disease, chronic obstructive pulmonary disease, heart disease, stroke, asthma, diabetes, HIV/AIDS, or morbid obesity, as estimated from the CDC 500 Cities Project (*16*), reported HIV prevalence (*17*), and reported morbid obesity prevalence (*18*,*19*), corrected for multiple conditions. The population of pregnant women is derived by using CDC's method combining fertility, abortion, and fetal loss rates (*20*–*22*).
